# Supplementary material for: Assessing capacities to strengthen intersectoral collaboration in Territorial Public Health Councils in the Republic of Moldova
Source: PLoS One. 2024 May 30;19(5):e0303821. doi: 10.1371/journal.pone.0303821 (PMC11139316; doi:10.1371/journal.pone.0303821)
Supplement: S1 File — (DOCX) [file pone.0303821.s001.docx]

**Supplement 1**

**S1 Fig 1** Distribution of respondents based on institutions represented in TPHC and members’ age (%)

Another important aspect is the members’ work experience. The majority of members, from various institutions, who are representatives at the Council have over 25 years of work experience (50%), with the majority (71,4%) being representatives of the Rayon Council. The minority is made of members with less than 10 years of experience, with an upward trend among members of group I - 22,2% work experience of TPHC presidents and secretaries. Structure of respondents based on work experience is as follows (Figure 2):

**S1 Fig 2** Structure of respondents based on work experience (%)


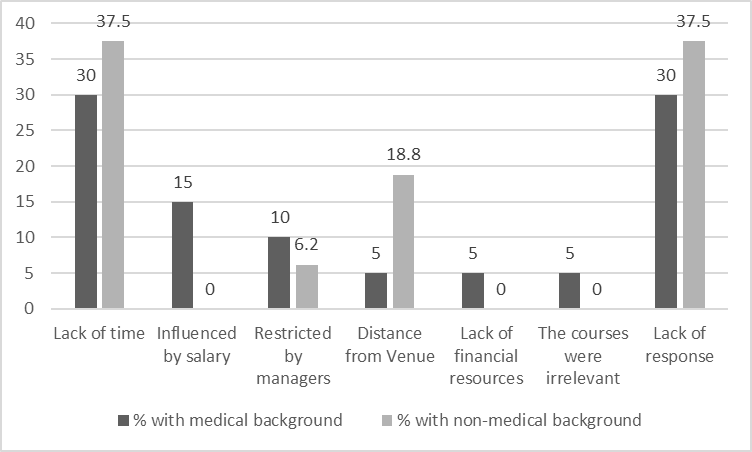


**S1 Fig 3** The main barriers to participation in training courses


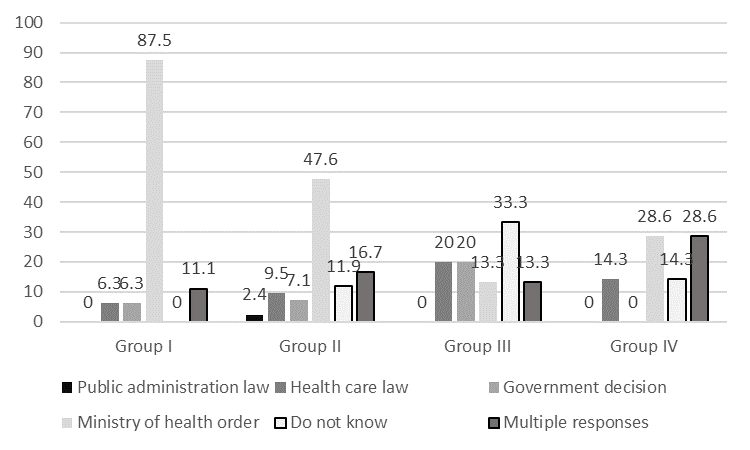


**S1 Fig 4** Knowledge of Law that regulates TPHC activity


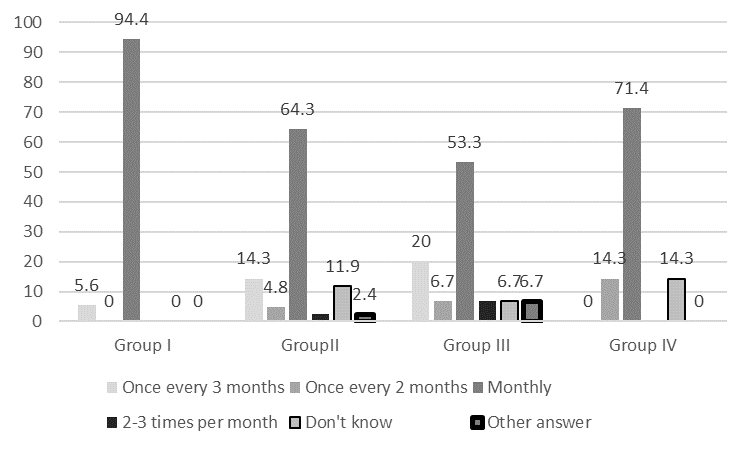


**S1 Fig 5** Rating the importance of monthly meetings
