# Supplementary material for: Assessing capacities to strengthen intersectoral collaboration in Territorial Public Health Councils in the Republic of Moldova
Source: PLoS One. 2024 May 30;19(5):e0303821. doi: 10.1371/journal.pone.0303821 (PMC11139316; doi:10.1371/journal.pone.0303821)
Supplement: S2 File — (DOCX) [file pone.0303821.s002.docx]

**Supplement 2**

**QUESTIONNAIRE: TERRITORIAL PUBLIC HEALTH COUNCIL (TPHC)**

**DEAR COLLEAGUE,**

In order to continuously improve the work of the Public Health Council at the territorial level, we highly appreciate your contribution. **We assure you of the confidentiality of this questionnaire.**

**THANK YOU FOR YOUR INVOLVEMENT IN THIS IMPORTANT ACTION!**

1. **Gender:** a. Female; b. Male.
2. **Your age:** a. under 40; b. 41 – 64; c. over 65.

**3. The institution you represent in the TPHC:**

1. The Chief State Sanitary Physician from the territory – Chairman of the Territorial Public Health Council;
2. The Secretary of the Territorial Public Health Council;
3. The Director of the PMSI Rayon Hospital;
4. The Head of the PMSI Health Center in the city of residence of the rayon (coordinator of primary medical care in the rayon);
5. The Head of the rayon pre-hospital emergency medical assistance substation;
6. The representative of private medical-sanitary/pharmaceutical and dentistry institutions;
7. The representative of the Rayon Council;
8. The representative of the Territorial Agency of the National Health Insurance Company;
9. The Chairman of the rayon trade union committee of the Trade Union Federation ‘Health’ in Moldova;
10. The representative of the Civil Protection and Emergency Situations Service;
11. The representative of the Territorial Department of Education;
12. The representative of the Ministry of Internal Affairs (Police);
13. The representative of the National Food Safety Agency (NFSA);
14. The representative of a local NGO;
15. The representative of the Environmental Service;
16. The representative of religious cults in the rayon;
17. The rayon media representative;
18. The representative of the rayon Social Assistance Service;
19. The representative of the rayon business environment;
20. The representative of the rayon Department of culture/art.
21. **Your professional experience:** a. less than 10 years b. 10-24 years c. more than 25 years
22. **Have there been any other mechanisms of cross-sector public health activities at rayon level prior to the implementation of the TPHC?**

a. Have not been b. Have been c. I do not know

1. **Are there policies, procedures, cross-sector collaboration processes in the field of public health at the territorial level?**
2. Yes
3. No
4. I do not know
5. **Do you have an approved plan on Council’s themes?**
6. Yes
7. No
8. I do not know
9. **When was the TPHC set up?**
   1. starting from 2016;
   2. starting from 2017;
   3. from the first half of 2018;
   4. from the second half of 2018;
   5. from the first half of 2019.
10. **Since when has the actual operation (concrete activities) of the TPHC started?**
    1. starting from 2016;
    2. starting from 2017;
    3. from the first half of 2018;
    4. from the second half of 2018;
    5. from the first half of 2019.
11. **How have cross-sector public health activities changed at rayon level since the implementation of TPHC?**

a. have changed a lot for the better;

b. have changed slightly for the better;

c. nothing has changed;

d. have changed slightly for the worse;

e. have changed a lot for the worse.

1. **If you answered A or B to the previous question (# 10), what has changed for the better?**
2. communication between all medical institutions in the rayon;
3. cooperation between all medical institutions in the rayon;
4. communication between all agencies represented in the TPHC in the rayon;
5. cooperation between all agencies represented in the TPHC in the rayon;
6. specialists from all sectors are better trained in the field;
7. better funding for rayon public health activities;
8. public health activities are more in number and more effective;
9. population health indicators have improved.
10. **How clear are the TPHC functions to you? Please circle the number on a scale from 0 to 10 (0 minimum – absolutely unclear, 10 maximum – absolutely clear)**

0 1 2 3 4 5 6 7 8 9 10

1. **How would you assess the TPHC’s capability in developing public health policies and setting priorities? Please circle the number on a scale from 0 to 10 (0 minimum, 10 maximum)**

0 1 2 3 4 5 6 7 8 9 10

1. **How clear is the TPHC’s activity strategy? Please circle the number on a scale from 0 to 10 (0 – absolutely unclear, 10 – absolutely clear)**

0 1 2 3 4 5 6 7 8 9 10

1. **How much does the activity in the TPHC influence the TPHC members’ motivation (including, financial one)? Please circle the number on a scale from 0 to 10 (0 – very little, 10 – very much)**

0 1 2 3 4 5 6 7 8 9 10

1. **Do you think that the fact that TPHC’s decisions are only advisable influences the quality of the activities? Please circle the number on a scale from 0 to 10 (0 – very little, 10 – very much)**

0 1 2 3 4 5 6 7 8 9 10

1. **Do you consider the support of the Ministry of Health, Labor and Social Protection in the TPHC’s work to be sufficient? Please circle the number on a scale from 0 to 10 (0 – very little, 10 – very much)**

0 1 2 3 4 5 6 7 8 9 10

1. **Do you consider the support of the National Public Health Agency (NPHA) in the TPHC’s work to be sufficient? Please circle the number on a scale from 0 to 10 (0 – very little, 10 – very much)**

0 1 2 3 4 5 6 7 8 9 10

1. **Do you consider the support of LPAs in the TPHC’s work to be sufficient? Please circle the number on a scale from 0 to 10 (0 – very little, 10 – very much)**

0 1 2 3 4 5 6 7 8 9 10

1. **Do you consider the support of external donors in the TPHC’s work to be sufficient? Please circle the number on a scale from 0 to 10 (0 – very little, 10 – very much)**

0 1 2 3 4 5 6 7 8 9 10

1. **Do you consider TPHC members’ theoretical knowledge on public health to be sufficient? Please circle the number on a scale from 0 to 10 (0 – very little, 10 – very much)**

0 1 2 3 4 5 6 7 8 9 10

1. **Do you consider TPHC members’ analytical skills on public health to be sufficient? Please circle the number on a scale from 0 to 10 (0 – very little, 10 – very much)**

0 1 2 3 4 5 6 7 8 9 10

1. **Do you consider the TPHC’s activity to be formal one? Please circle the number on a scale from 0 to 10 (0 – completely disagree, 10 – completely agree)**

0 1 2 3 4 5 6 7 8 9 10

1. **Do you think that the number of TPHC members is sufficient?**

A. Yes B. No, too many C. No, too few.

1. **Do you think that the TPHC’s members represent all the authorities responsible for Public Health in your rayon?**

A. Yes, there are all necessary authorities.

B. No, other authorities/members of society should also be invited to be members of the TPHC

1. **If you answered B, who else (please circle):**
2. a representative of the Ministry of Internal Affairs (Police);
3. a representative of the NFSA;
4. a representative of a local NGO;
5. a representative of the Environmental Service;
6. a representative of religious cults in the rayon;
7. a rayon media representative;
8. a representative of the rayon Social Assistance Service;
9. a representative of the rayon business environment;
10. a representative of the rayon Department of culture/art.
11. Others, please specify ________________________
12. ________________________
13. ________________________
14. **Do you think that the management of the TPHC ensured by the representatives of the Public Health Center is appropriate and effective?**
15. Yes;
16. No, management should be ensured by the representatives of LPAs;
17. No, management should be ensured by the representatives of the Rayon Hospital;
18. No, management should be ensured by the representatives of Primary Health Care;
19. No, management should be ensured by highly authoritative representatives;
20. No, management should be ensured by rotation by all members of the TPHC;
21. No, management should be elected by vote of the TPHC’s members;
22. Other variant ___________________________________________________
23. **If you think that the management of the TPHC ensured by the representatives of the Public Health Center is not appropriate and effective, what are the main causes?**
24. insufficient authority / there are no true leaders;
25. are retired and/or of the old school;
26. do not have enough modern knowledge about public health;
27. do not have sufficient communication skills;
28. have no levers of influence;
29. have no independence in decision-making;
30. do not have the necessary financial resources;
31. do not have clear visions;
32. is not supported by LPAs.
33. **What are the most active representatives in the TPHC in your rayon (please circle):**
34. Chief State Sanitary Physician from the territory – Chairman of the Territorial Public Health Council;
35. The Secretary of the Territorial Public Health Council;
36. The Director of the PMSI Rayon Hospital;
37. The Head of the PMSI Health Center in the city of residence of the rayon (coordinator of primary health care in the rayon);
38. The Head of the rayon pre-hospital emergency medical assistance substation;
39. The representative of private medical-sanitary/pharmaceutical and dentistry institutions;
40. The representative of the Rayon Council;
41. The representative of the Territorial Agency of the National Health Insurance Company;
42. The Chairman of the rayon trade union committee of the Trade Union Federation ‘Health’ in Moldova;
43. The representative of the Civil Protection and Emergency Situations Service;
44. The representative of the Territorial Department of Education;
45. The representative of the Ministry of Internal Affairs (Police);
46. The representative of the NFSA;
47. The representative of a local NGO;
48. The representative of the Environmental Service;
49. The representative of religious cults in the rayon;
50. The rayon media representative;
51. The representative of the rayon Social Assistance Service;
52. The representative of the rayon business environment;
53. The representative of the rayon Department of culture/art.
54. **Do you think that within the TPHC do you have obvious leaders in the field of health promotion (no matter what sector he/she comes from)?**
55. Yes
56. No
57. I am not sure
58. **How would you characterize the relationships with the colleagues from TPHC?**

**a.** Team work;

**b.** Collegial collaboration;

**c.** Good ones, but with slight misunderstandings;

**d.** Strained;

**e.** Conflictual.

1. **How do you think what is/would be the main organization engaged in cross-sector public health actions at the territorial level?**
2. NPHA / PHC
3. PMSI Rayon Hospital
4. PMSI Health Center
5. Rayon Councils
6. Others ___________
7. **How would you assess the importance of members (and those who you would like to be part of) in the TPH Council’s work and in promoting public health activities in the rayon? Please circle the number on a scale from 0 to 10 (0 minimum, 10 maximum)**

|  | IMPORTANCE  of TPHC’s members | 0 | 1 | 2 | 3 | 4 | 5 | 6 | 7 | 8 | 9 | 10 |
| --- | --- | --- | --- | --- | --- | --- | --- | --- | --- | --- | --- | --- |
|  | the Chief State Sanitary Physician from the territory; |  |  |  |  |  |  |  |  |  |  |  |
|  | the Secretary of the Territorial Public Health Council; |  |  |  |  |  |  |  |  |  |  |  |
|  | the Director of the PMSI Rayon Hospital; |  |  |  |  |  |  |  |  |  |  |  |
|  | the Coordinator of primary medical care in the rayon; |  |  |  |  |  |  |  |  |  |  |  |
|  | the Head of the rayon pre-hospital emergency medical assistance substation; |  |  |  |  |  |  |  |  |  |  |  |
|  | the representative of private medical-sanitary/pharmaceutical and dentistry institutions; |  |  |  |  |  |  |  |  |  |  |  |
|  | the representative of the Rayon Council; |  |  |  |  |  |  |  |  |  |  |  |
|  | the representative of the Territorial Agency of the National Health Insurance Company; |  |  |  |  |  |  |  |  |  |  |  |
|  | the Chairman of the rayon trade union committee of the Trade Union Federation ‘Health’ in Moldova; |  |  |  |  |  |  |  |  |  |  |  |
|  | the representative of the Civil Protection and Emergency Situations Service; |  |  |  |  |  |  |  |  |  |  |  |
|  | the representative of the Territorial Department of Education; |  |  |  |  |  |  |  |  |  |  |  |
|  | the representative of the Ministry of Internal Affairs (Police); |  |  |  |  |  |  |  |  |  |  |  |
|  | the representative of the NFSA; |  |  |  |  |  |  |  |  |  |  |  |
|  | the representative of a local NGO; |  |  |  |  |  |  |  |  |  |  |  |
|  | the representative of the Environmental Service; |  |  |  |  |  |  |  |  |  |  |  |
|  | the representative of religious cults in the rayon; |  |  |  |  |  |  |  |  |  |  |  |
|  | the rayon media representative |  |  |  |  |  |  |  |  |  |  |  |
|  | the representative of the rayon Social Assistance Service |  |  |  |  |  |  |  |  |  |  |  |
|  | the representative of the rayon business environment; |  |  |  |  |  |  |  |  |  |  |  |
|  | the representative of the rayon Department of culture/art |  |  |  |  |  |  |  |  |  |  |  |

1. **Do you consider the number of TPHC’s decisions and joint orders, issued throughout the activity, to be relevant to the time and degree of public health issues in your rayon?**

a. It is much smaller than we could have accomplished;

b. It is smaller than we could have accomplished;

c. It is exact of what we could have accomplished;

d. It is quite large for the period of activity;

e. It is too large for the period of activity.

1. **What are the most examined territorial public health problems?**
2. Cooperation between medical institutions and LPAs in the territory;
3. Issues regarding the observance of the regulations and laws in force;
4. Health problems of the population in the territory and its determinants;
5. National and Territorial Health Programs;
6. Public health emergencies in the territory.
7. **How prepared is the Territorial Public Health Council for performing the following duties? Please circle the number on a scale from 0 to 10 (0 absolutely unprepared – 10 very prepared)**

|  | Areas of responsibility | 0 | 1 | 2 | 3 | 4 | 5 | 6 | 7 | 8 | 9 | 10 |
| --- | --- | --- | --- | --- | --- | --- | --- | --- | --- | --- | --- | --- |
|  | Examining the rayon health system’s current organization and operation issues; |  |  |  |  |  |  |  |  |  |  |  |
|  | Ensuring collaboration between medical or public medical-sanitary institutions in the territory |  |  |  |  |  |  |  |  |  |  |  |
|  | Developing, coordinating and promoting territorial public health programs, and exercising control over their implementation; |  |  |  |  |  |  |  |  |  |  |  |
|  | Resolving the health problems of the population in the territory and their determinants; |  |  |  |  |  |  |  |  |  |  |  |
|  | Ensuring implementation of national and territorial health programs; |  |  |  |  |  |  |  |  |  |  |  |
|  | Implementing and analyzing territorial Health Profiles |  |  |  |  |  |  |  |  |  |  |  |
|  | Ensuring implementation of policies in the field of human resources for health |  |  |  |  |  |  |  |  |  |  |  |
|  | Organizing and exercising complex measures of response and control in public health emergencies |  |  |  |  |  |  |  |  |  |  |  |
|  | Involving the society in provision of public health services; |  |  |  |  |  |  |  |  |  |  |  |
|  | Supporting development of the quality drinking water supply plans, protecting free air, ensuring waste removal and sanitation |  |  |  |  |  |  |  |  |  |  |  |
|  | Coordinating the allocation of financial, including investment, resources in the field of state surveillance of public health; |  |  |  |  |  |  |  |  |  |  |  |
|  | Facilitating the organization of sociological studies on the quality of healthcare services provided in the territory, screening of the population health in the administrative territory; |  |  |  |  |  |  |  |  |  |  |  |
|  | Measuring the results and collecting feedback for policies adjustment |  |  |  |  |  |  |  |  |  |  |  |
|  | Ensuring accountability of all relevant stakeholders |  |  |  |  |  |  |  |  |  |  |  |

1. **How do you appreciate the provision with the information and materials needed for you to do the job properly? Please circle the number on a scale from 0 to 10 (0 – insufficient, 10 – sufficient)**

0 1 2 3 4 5 6 7 8 9 10

1. **Is it easy for you to communicate within the TPHC? Appreciate the level of communication, by circling the number on a scale from 0 to 10 (0 – minimum, 10 – maximum):**

0 1 2 3 4 5 6 7 8 9 10

1. **Specify the legislative act that regulates the TPHC’s activity:**
2. Law on public administration;
3. Law on health protection;
4. Government Decision;
5. Order of the Ministry of Health, Labor and Social Protection;
6. I do not know.

1. **Have you personally familiarized yourself with the legislative act that regulates the TPHC’s activity?**
2. Yes;
3. No;
4. Partially only.
5. **How many times does the TPHC meet normally?**
6. once every 3 months;
7. once every 2 months;
8. once a month;
9. -3 times per month;
10. I do not know.
11. **In the past 3 years, have you finished/attended public health courses?**

**a**. Yes **b**. No

1. **If you answered ‚Yes’, where? (choose one or more variants):**
2. Courses promoted by MoHLSP;
3. Continuous training courses/specialized programs promoted by SUMP ‚Nicolae Testemitanu’;
4. Continuous training courses/specialized programs promoted by other national institutions;
5. Courses promoted by the ‚Healthy Life’ project;
6. Courses held outside the Republic of Moldova.
7. **If you answered ‚No’, why? (choose one or more variants):**

**a**. Lack of time;

**b.** Courses are formal;

**c.** Lack of financial resources;

**e.** Distance from the place where the courses are held;

**f.** Lack of influence on salary;

**g.** Restricted by top managers;

**h.** Courses are insignificant/irrelevant.

1. **Do you think that you need to get informed on public health in order to work effectively within the TPHC?**
   1. Yes, I urgently need to receive large amounts of information;
   2. Yes, I need to get informed on certain segments;
   3. Yes, I only need to get informed on newer things in the field;
   4. I do not need, I have all the necessary knowledge;
   5. I do not need, I am not interested in, as it is not my direct interest/direct responsibility.
2. **Are there knowledge and skills to initiate, develop and implement disease prevention and health promotion programs at rayon level and to measure change? Please circle the number on a scale from 0 to 10 (0 – very few, 10 – a great deal)**

0 1 2 3 4 5 6 7 8 9 10

1. **Are there knowledge and skills to influence the social determinants of health in order to accelerate improvements for the most vulnerable people? Please circle the number on a scale from 0 to 10 (0 – very few, 10 – a great deal)**

0 1 2 3 4 5 6 7 8 9 10

1. **Are there examples of good practices of cross-sector actions for disease prevention and health promotion, social determinants of health and/or of health promotion in all policies?**
2. Yes
3. No
4. I do not know
5. **How important/useful is the monthly meeting of the Council? Please circle the number on a scale from 0 to 10 (0 – very little, 10 – very much)**

0 1 2 3 4 5 6 7 8 9 10

1. **Is there a tool for assessing the Council’s capabilities and approved decisions?**
2. Yes
3. No
4. I do not know
5. **How would you assess the importance of barriers in the TPHC’s activity and in promoting public health activities in the rayon? Please circle the number on a scale from 0 to 10 (0 – minimum, 10 – maximum)**

|  | IMPORTANCE  of barriers in TPHS’s activity | 0 | 1 | 2 | 3 | 4 | 5 | 6 | 7 | 8 | 9 | 10 |
| --- | --- | --- | --- | --- | --- | --- | --- | --- | --- | --- | --- | --- |
|  | Lack of sufficient human resources; |  |  |  |  |  |  |  |  |  |  |  |
|  | Lack of competent human resources; |  |  |  |  |  |  |  |  |  |  |  |
|  | Lack of financial resources; |  |  |  |  |  |  |  |  |  |  |  |
|  | Lack of information resources; |  |  |  |  |  |  |  |  |  |  |  |
|  | Lack of theoretical and practical knowledge; |  |  |  |  |  |  |  |  |  |  |  |
|  | Insufficient communication at rayon level; |  |  |  |  |  |  |  |  |  |  |  |
|  | Insufficient communication at inter-rayon level; |  |  |  |  |  |  |  |  |  |  |  |
|  | Insufficient communication at rayon and central level; |  |  |  |  |  |  |  |  |  |  |  |
|  | Low level of responsibility of TPHC’s members; |  |  |  |  |  |  |  |  |  |  |  |
|  | Insufficient control over TPHC’s activity; |  |  |  |  |  |  |  |  |  |  |  |
|  | Insufficient involvement of the population in TPHC’s activity; |  |  |  |  |  |  |  |  |  |  |  |
|  | Insufficient legislative-normative framework for TPHC’s activity; |  |  |  |  |  |  |  |  |  |  |  |
|  | Insufficient financial motivation of TPHC’s members; |  |  |  |  |  |  |  |  |  |  |  |
|  | Insufficient support from LPAs. |  |  |  |  |  |  |  |  |  |  |  |

1. **How do you appreciate prospects for the development of the Territorial Public Health Council in your rayon? Please circle the number on a scale from 0 to 10 (0 – very weak, 10 – very strong)**

0 1 2 3 4 5 6 7 8 9 10

**Thank you for your contribution.**
