# Supplementary material for: Assessing capacities to strengthen intersectoral collaboration in Territorial Public Health Councils in the Republic of Moldova
Source: PLoS One. 2024 May 30;19(5):e0303821. doi: 10.1371/journal.pone.0303821 (PMC11139316; doi:10.1371/journal.pone.0303821)
Supplement: S3 File — (DOCX) [file pone.0303821.s003.docx]

**Supplement 3**

In developing the research, the Interview Guide was applied, presented below:

- Transition questions

Why do people get sick? Who bears the responsibility?

- Key questions

Appreciation of the level of knowledge and skills in the field of health of the population in the district,

- Name a health problem that you consider a priority for the population in your district. How did you find out about her? What do you know about this disease?
- What would you like to see done in this regard? Are you personally involved in solving it?
- Who should be the partners / actors involved?
- Do you think you are sufficiently informed about the state of health of the population of your district?

I. Identification of the degree of involvement of interested parties, partnerships and collaboration mechanisms

- Do you think that in your district they pay enough attention to the health problem you have identified?
- Are there improvements? Who sees/measures them and how? Who do you think should monitor this process?

II. Perception of TCPH role, responsibility and obligations by members; institutional mechanisms and the achievement of leadership.

- How did you find out that you are a member of the Council? What reaction did you have when you were informed about the creation of this Council? Why?
- What do you know about your rights and obligations as a member of this Council? Who informed you and how? Would you like to know something more? How would you prefer to be informed?
- Describe the feeling you have when you are invited to the Council meeting. .

III. Councils' capacities to collect and synthesize data to create a vision; determining the ability to formulate objectives and set priorities, manage human and financial resources, as well as the ability to develop an appropriate process for monitoring and adjusting policies.

- How and who determines the agenda of Council meetings? How do you find out about her?
- When a public health problem is determined, who distributes responsibilities? Do you agree with this procedure?
- Name 2 strong points and 2 weak points that you determine in the activity of your Council. What would be the possibility of strengthening the weak points identified?
- Give an example of success in addressing a health problem in your district. Why did it go?
- Give an example of an insufficient, unsuccessful approach to a public health problem. Why didn't it work? Appreciate the Council's role in this.
- Did these experiences have any impact on the development of subsequent policies/programmes? What are the lessons learned?
- Who checks and monitors the activity of the Council? Appreciate the efficiency and necessity of this process.
- Closing questions
- If today were a jubilee since the creation of the Council, what would you like for this body?
- Of all the issues mentioned in the discussion, which is the most important for you?
- Summarizing: a 2-3 min conclusion is made through a synthesis of what was discussed
- Do you agree? How well did I understand the issues you presented? Did I miss something? Is there something you haven't had a chance to express?

Basic techniques for supporting the discussion: the pause of a few seconds. after the involvement of each participant, insistence: we ask for details, development of the subject, explanation, example, continuation of the thought.

At the end of the discussion (summarization), about 10-15 minutes were provided to give the participants the opportunity to add information, aspects not touched upon during the interview or not foreseen in the guide.

The interviews were led by the moderator, and two other researchers participated as observers, also monitoring the non-verbal behavior of the participants.

The interviews were recorded, with the consent of the participants, and were later transcribed and coded.
