# Supplementary material for: Assessing capacities to strengthen intersectoral collaboration in Territorial Public Health Councils in the Republic of Moldova
Source: PLoS One. 2024 May 30;19(5):e0303821. doi: 10.1371/journal.pone.0303821 (PMC11139316; doi:10.1371/journal.pone.0303821)
Supplement: S5 File — (DOCX) [file pone.0303821.s005.docx]

**Supplement 5**

**Table 1** Perceptions of importance of members for TPHC activity

|  | **Members / participants** | **Importance** |
| --- | --- | --- |
| 1 | TPHC President | 9,2 |
| 2 | Secretary | 9,2 |
| 3 | PMSI RH Director | 8,8 |
| 4 | Head of PMSI HS | 8,8 |
| 5 | Head of EHH | 8 |
| 6 | Representative of Private MSI | 7 |
| 7 | Representative of District Council | 9 |
| 8 | Representative of CNAM | 7,5 |
| 9 | Representative of the ,,Sanatatea” Sindicate | 7,1 |
| 10 | Representative of CP and ES Service | 7,3 |
| 11 | Representative of Education Department | 7,9 |
| 12 | Representative of MIA | 7,2 |
| 13 | Representative of NAFS | 7,1 |
| 14 | Representative of local NGO | 6,4 |
| 15 | Representative of Ecology Service | 6,8 |
| 16 | Representative of religious cults from the district | 5,5 |
| 17 | Representative of local mass-media | 6,9 |
| 18 | Representative of social assistance service from the district | 7,4 |
| 19 | Representative of the business environment from the district | 5,7 |
